# Synthesis and In Vitro Characterization of Ascorbyl Palmitate-Loaded Solid Lipid Nanoparticles

**DOI:** 10.3390/polym14091751

**Published:** 2022-04-26

**Authors:** Maja Ledinski, Ivan Marić, Petra Peharec Štefanić, Iva Ladan, Katarina Caput Mihalić, Tanja Jurkin, Marijan Gotić, Inga Urlić

**Affiliations:** 1 Faculty of Science, Division of Molecular Biology, Department of Biology, University of Zagreb, 10 000 Zagreb, Croatia; maja.ledinski@biol.pmf.hr (M.L.); petra.peharec.stefanic@biol.pmf.hr (P.P.Š.); katarina.caput.mihalic@biol.pmf.hr (K.C.M.); 2 Division of Materials Chemistry, Radiation Chemistry and Dosimetry Laboratory, Ruđer Bošković Institute, 10 000 Zagreb, Croatia; imaric@irb.hr (I.M.); tanja.jurkin@irb.hr (T.J.); 3 Croatian Institute of Public Health, Department of Health Ecology, 10 000 Zagreb, Croatia; iva.ladan@hzjz.hr; 4 Division of Materials Physics, Laboratory for Molecular Physics and Synthesis of New Materials, Ruđer Bošković Institute, 10 000 Zagreb, Croatia; gotic@irb.hr

**Keywords:** ascorbate, ascorbyl palmitate, drug delivery, cellular uptake, nanoparticles, antitumor effect

## Abstract

Antitumor applications of ascorbic acid (AA) and its oxidized form dehydroascorbic acid (DHA) can be quite challenging due to their instability and sensitivity to degradation in aqueous media. To overcome this obstacle, we have synthesized solid lipid nanoparticles loaded with ascorbyl palmitate (SLN-AP) with variations in proportions of the polymer Pluronic F-68. SLNs were synthesized using the hot homogenization method, characterized by measuring the particle size, polydispersity, zeta potential and visualized by TEM. To investigate the cellular uptake of the SLN, we have incorporated coumarin-6 into the same SLN formulation and followed their successful uptake for 48 h. We have tested the cytotoxicity of the SLN formulations and free ascorbate forms, AA and DHA, on HEK 293 and U2OS cell lines by MTT assay. The SLN-AP in both formulations have a cytotoxic effect at lower concentrations when compared to ascorbate applied the form of AA or DHA. Better selectivity for targeting tumor cell line was observed with 3% Pluronic F-68. The antioxidative effect of the SLN-AP was observed as early as 1 h after the treatment with a small dose of ascorbate applied (5 µM). SLN-AP formulation with 3% Pluronic F-68 needs to be further optimized as an ascorbate carrier due to its intrinsic cytotoxicity.

## 1. Introduction

**Figure 1:**
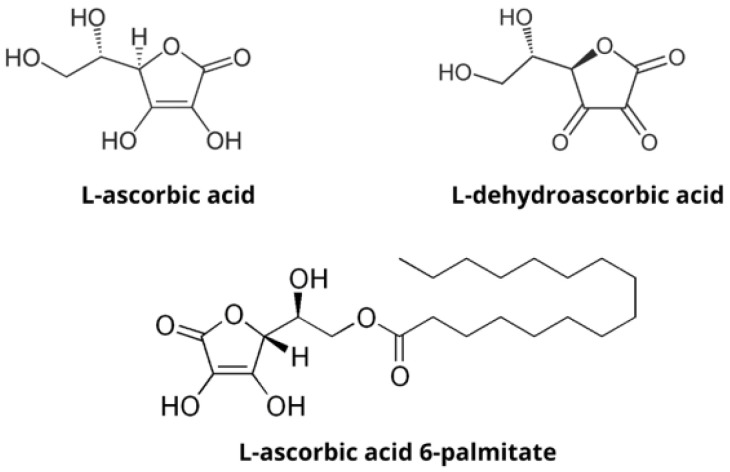
Chemical structures of hydrophilic compounds ascorbic acid and dehydroascorbic acid and lipophilic derivative, ascorbyl palmitate.

Ascorbate has been extensively studied as an antitumor agent, but its instability remains the major problem for its use. Ascorbic acid (AA) and its oxidized form, dehydroascorbic acid (DHA), are readily degraded in aqueous media in the presence of oxygen and metal ions and are also sensitive to light. To improve the stability of ascorbate, salts and lipophilic derivatives of AA are often used [1]. The alkyl esters of AA are of particular interest because they can penetrate cell membranes, however, they are poorly soluble in aqueous environments [2]. Ascorbyl palmitate (Figure 1) is an alkyl ester already used in the cosmetics, dermatology, and food industries as a more stable lipophilic derivative of AA because it retains the antioxidant properties of ascorbate [3]. Therefore, it is important to develop formulations that could allow its administration under hydrophilic conditions.

In order to apply AP in hydrophilic conditions, researchers have already tried various formulations. Kristl et al. [4] tested the incorporation of AP into microemulsions, liposomes, and solid lipid nanoparticles (SLNs), which were evaluated as the second most stable out of five formulations. On the other hand, Gopinath et al. [5] developed a method for the formation of ascorbyl palmitate bilayer vesicles (aspasomes) using the film hydration method and sonication. They had found that AP could assemble into bilayer vesicles by adding cholesterol and diacetyl phosphate. Teeranachaideekul et al. [6] also investigated the incorporation of AP into lipid nanoparticles by synthesizing both SLN and nanostructured lipid carriers by high-pressure homogenization. They found that AP in nanoparticles with glycerol monostearate produced stable formulations with high (100%) encapsulation efficiency. 

Ascorbate is a known antioxidant, however, depending on the concentration it can have a prooxidant effect that selectively affects tumor cells [7]. Although this antitumor effect is mostly explained by prooxidant activity of ascorbate, it can also affect tumor cells by inducing epigenetic modifications, affecting glycolysis metabolism and surviving in hypoxia [7,8]. Therefore, AP, as a carrier of ascorbate, was recognized as an antitumor agent and researchers have incorporated it into nanoparticles with various chemotherapeutic agents. Zhou et al. [9] incorporated AP and paclitaxel into the SLN, Li et al. [10] synthesized liposomes for co-administration of AP and docetaxel, and Jukanti et al. [11] PEGylated liposomes for co-administration of AP and doxorubicin. All of these formulations were designed to combine the antitumor activity reported for ascorbate with the cytotoxic effect of chemotherapeutic agents, which is referred to as combination chemotherapy.

**Figure 2:**
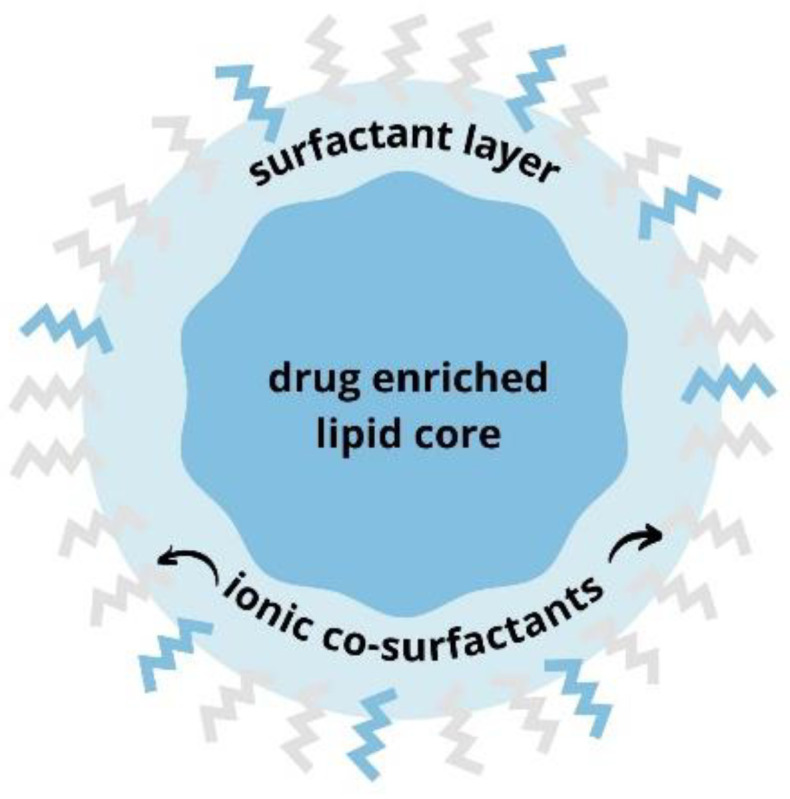
The structure of solid lipid nanoparticle. SLNs have lipid core with an incorporated lipid compound of interest and a surfactant layer on the surface which enables solubility in hydrophilic solutions. Ionic co-surfactants provide electrical stability and prevent the possible aggregation of nanoparticles.

Several sources indicate that the polymers that go into these formulations must be carefully selected because of their effect on the stability of the nanoparticle formulations [5,11]. In this work, we synthesized and characterized ascorbyl palmitate-loaded SLNs (Figure 2) with different proportions of the polymer Pluronic F-68, which is commonly used as a surfactant in the synthesis of nanoparticles due to its amphiphilic properties and good biocompatibility [12]. 

In the study of drug delivery systems, confirmation of cellular uptake is crucial before further experiments are performed. We followed nanoparticle uptake by visualizing the uptake of SLN loaded with fluorescent coumarin-6. We also analyzed the cytotoxicity of SLN on HEK 293 and U2OS cell lines to test whether these formulations could have an antitumor potential.

## 2. Materials and Methods

### 2.1. Materials

Glyceryl monostearate (GMS), dimethyldioctadecylammonium bromide (DDAB), ascorbyl palmitate (AP), ascorbic acid (AA), dehydroascorbic acid (DHA), coumarin-6, MTT (3-(4,5-Dimethylthiazol-2-yl)-2,5-Diphenyltetrazolium Bromide), 0.25% Trypsin/EDTA, 2′,7′-Dichlorofluorescin diacetate and Pluronic F-68 were purchased from Sigma Aldrich, Germany. Dulbecco’s Modified Eagle Medium High glucose (4.5 g/L), fetal bovine serum (FBS) and penicillin/streptomycin were purchased from Capricorn, Germany.

### 2.2. SLN Synthesis and Characterization

**Figure 3:**
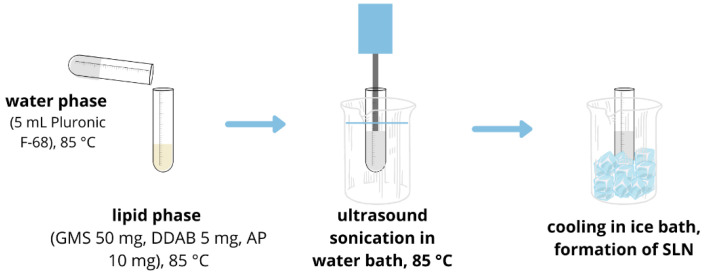
Synthesis of solid lipid nanoparticles.

Ascorbyl palmitate-loaded SLNs were prepared by ultrasonic method [9]. GMS (50 mg), DDAB (5 mg) and AP (10 mg) were heated to 85 °C, just above the melting point of GMS. At the same time, 5 mL of Pluronic-F68 (either 3% or 10%) was heated to the same temperature, in a water bath. When both phases reached 85 °C, an aqueous phase was added to the lipid phase and pre-emulsion was sonicated for 25 min in a water bath at 85 °C (Figure 3). SLNs were formed after cooling in an ice bath and were stored at 4 °C. Size, polydispersity and zeta potential of SLNs were measured by multiple angle dynamic light scattering (MADLS) using Zetasizer Ultra (Malvern Panalytical). The size distributions given by MADLS were reported as distributions by intensity, and results are presented as the mean value of at least 3 measurements each, performed at three angles. Zeta potential was taken as the mean value of three measurements. For visualization using TEM, SLNs were firstly incubated on a Formvar^®^/carbon copper grids and washed in ultra-pure water (Milli-Q, 18.2 MΩ·cm, Merck Millipore, Billerica, MA, USA). Sample was then incubated with uranyl acetate for 5 min and after staining washed in ultra-pure water 3 times. After the sample had dried, SLNs were visualized using a transmission electron microscope Morgagni 268D (Philips/FEI) and operating at 70 kV.

### 2.3. Cell Culture

Experiments were performed using human embryonic kidney HEK 293 and human osteosarcoma U2OS cell lines, which were kindly provided by Vjekoslav Tomaić (Ruđer Bošković Institute, Zagreb, Croatia). HEK 293 and U2OS cell lines were cultured in DMEM High glucose (4.5 g/L) supplemented with 10% FBS and 1% penicillin/streptomycin. Cells were cultured at 37 °C in the presence of 5% CO_2_.

### 2.4. Cellular Uptake of Solid Lipid Nanoparticles

SLNs containing coumarin-6 were synthesized using the same method as mentioned above. Synthesis was carried out in the dark. To remove free coumarin-6 from the SLN suspension, SLN-coumarin-6 were centrifuged at 20,000× *g*, 30 min (Hettich universal 320R) and SLN-coumarin-6 pellet was resuspended in ultra-pure water. Cells were seeded at a density of 30,000 cells/mL in a Petri dish and allowed to adhere overnight at 37 °C, 5% CO_2_. They were treated with SLN-coumarin-6 and visualized by using fluorescent microscopy (Olympus BX51; software DPController, Olympus Optical) at time points 1 h, 3 h, 6 h, 24 h and 48 h.

### 2.5. Cytotoxicity Analysis Using MTT Test

HEK 293 and U2OS cells were seeded in 96-well plate (1 × 10^4^ cells/well) and allowed to adhere overnight at 37 °C, 5% CO_2_. Treatments (SLN-AP, blank SLN, AA and DHA) were prepared in cell culture medium. Cells were treated with blank SLNs in the same amount as SLN-AP for each concentration to serve as a control for the effect of the empty nanoparticles. In addition, all treatment concentrations were normalized to ascorbate composition to account for differences in molecular masses between AA, DHA and AP. Cells were treated in triplicates and after 24 h, treatment medium was aspirated, and cells were washed three times with 200 μL PBS. Cells were incubated 4 h with MTT solution in cell culture medium at concentration 0.5 mg/mL, and 170 μL DMSO was added to dissolve formazan crystals. Absorbance was measured at 570 nm using GloMax microplate reader (Promega), and cell viability was calculated as a percentage of untreated control after reducing blank absorbance to all the samples.

### 2.6. Analysis of Reactive Oxygen Species

U2OS cells were seeded in black 96-well plates (2 × 10^4^ cells/well) and incubated for 24 h at 37 °C, 5% CO_2_. Treatments (AA in free form, SLN-AP, blank SLN) were prepared in cell culture medium and cells were treated in triplicates for 1 h and 6 h. After the incubation, cells were washed 3 times with PBS to remove any treatment residue. Then, 20 µM 2′,7′-dichlorofluorescin diacetate (DCHF-DA) was prepared in PBS and cells were incubated for 30 min at 37 °C, 5% CO_2_. Fluorescence was measured at excitation 490 nm, emission 510–570 nm using GloMax microplate reader (Promega).

## 3. Results

### 3.1. SLN Synthesis and Characterization

**Figure 4:**
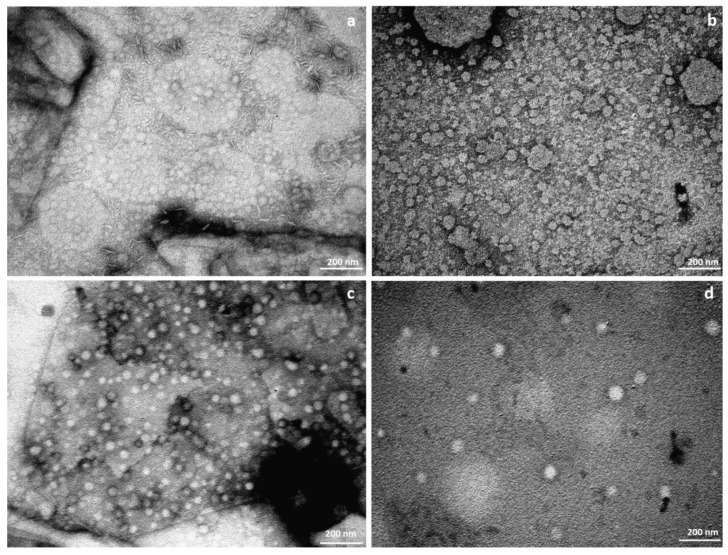
TEM images of SLN. SLN suspensions were contrasted with uranyl acetate and washed in ultra-pure water before visualization. Scale bar = 200 nm (**a**) SLN-AP 3% Pluronic; (**b**) blank SLN 3% Pluronic; (**c**) SLN-AP 10% Pluronic; and (**d**) blank SLN 10% Pluronic.

The SLN were characterized by measuring their size, polydispersity and zeta potential. All nanoparticles have nanometer size as shown in Table 1 and Figure 4. SLN-APs prepared in 3% Pluronic are larger in size and have a smaller polydispersity index when compared to blank SLNs of the same formulation, which are smaller in size but have a higher polydispersity index. The SLN-APs prepared in 10% Pluronic are smaller than the blank SLNs of the same formulation, but the polydispersity indices do not differ greatly. Both formulations of the empty SLNs have a positive zeta potential, while SLN-APs have a very low positive for 3% Pluronic F-68 formulation and slightly negative zeta potential for 10% Pluronic F-68 formulation.

Morphological evaluation of all the SLN suspensions using TEM revealed that both suspensions contain round SLNs with a size in the nanometer range. Rod-like shapes are seen in the formulation SLN-AP (Figure 4a) prepared with 3% Pluronic. The image of empty SLNs prepared in 3% Pluronic (Figure 4b) confirms the polydispersity data with different sizes of SLNs. Likewise, the images of SLNs prepared in 10% Pluronic (Figure 4c,d) are consistent with the DLS data.

### 3.2. Cellular Uptake of SLN

**Figure 5:**
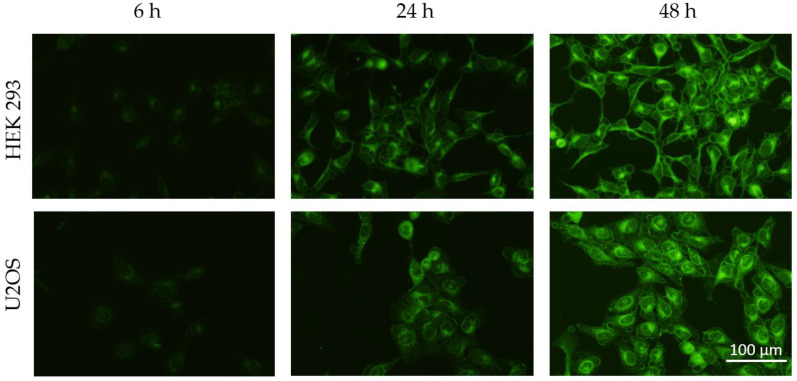
Cellular uptake of SLN-coumarin-6 during 48 h in cell lines HEK 293 and U2OS. Cells were treated with SLN-coumarin-6 and photographed at time points 6 h, 24 h and 48 h. Green fluorescence represents coumarin-6 that entered the cells. Scale bar = 100 µm.

HEK 293 and U2OS cell lines were incubated with SLN-coumarin-6 and visualized under a fluorescence microscope. Fluorescence is detected in both cell types and increases with incubation time, as shown in Figure 5.

### 3.3. Cytotoxicity Analysis

We used the MTT assay to evaluate the cytotoxicity of the two SLN formulations, prepared in 3% and 10% Pluronic F-68.

**Figure 6:**
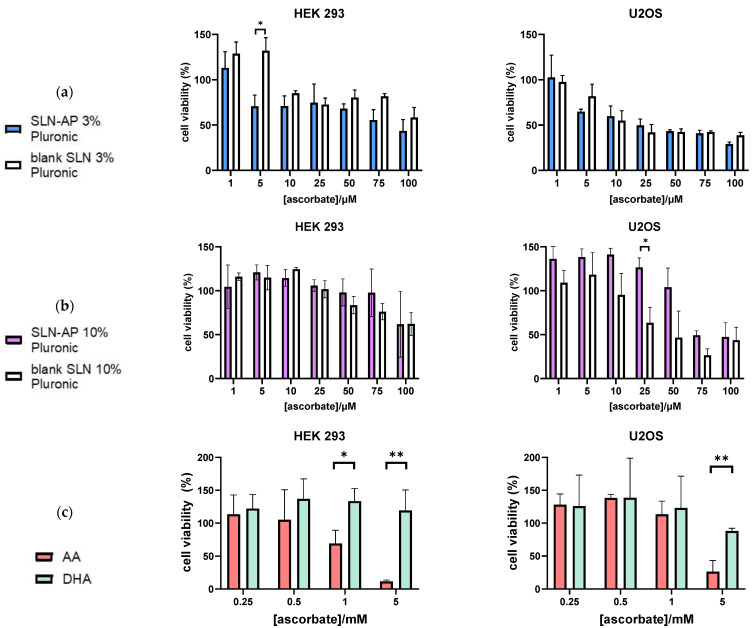
Cytotoxicity of SLN AP and blank SLN synthesized with either 3% (**a**) or 10% (**b**) Pluronic F-68, as well as AA and DHA in free form (**c**), on HEK 293 and U2OS cell lines was tested by MTT test during 24 h. All treatments were normalized to ascorbate composition. Data are expressed as percentage of negative control, mean ± sd, *n* = 3. Holm–Šídák multiple unpaired t-tests were performed. Statistical significance: * *p* ≤ 0.05, ** *p* ≤ 0.01.

Regarding the SLN prepared in 3% Pluronic F-68, blank SLN presented mild cytotoxicity in HEK 293 cells, and stronger cytotoxicity in tumor U2OS cells. Likewise, SLN-AP also presented mild cytotoxicity on HEK 293 cell and stronger in U2OS cells. For concentrations higher than 5 µM of ascorbate, SLN-AP show cytotoxic effect on both cell lines, HEK 293 and U2OS, but the data suggest that tumor cells are slightly more sensitive to the treatment (Figure 6a).

When analyzing data regarding the SLN prepared in 10% Pluronic F-68, blank SLN presented selective cytotoxicity in HEK 293 and U2OS cell, significantly reducing viability in tumor U2OS cell. After 50 µM ascorbate, SLN-AP of this formulation begin to exert a cytotoxic effect, as shown in Figure 6b. U2OS cell are more sensitive to the treatment with SLN-AP then HEK293 cells (Figure 6b).

Altogether, cytotoxicity is higher for the SLN prepared with 3% Pluronic F-68, and tumor cells are slightly more sensitive to the SLN and SLN-AP treatments than HEK 293.

To evaluate the difference in cytotoxicity between the SLN formulations and ascorbate in AA and DHA, we performed a cytotoxicity assay and treated the cells in the same way with AA and DHA. While DHA shows no cytotoxic effect on HEK 293 and U2OS cell lines, AA has an IC50 of 1.94 mM for HEK 293 and 4.17 mM for U2OS, as shown in Figure 6c. Therefore, SLN-APs, prepared in 3% and 10% Pluronic, are effective in much lower concentrations than AA and DHA in the free form.

### 3.4. Analysis of Reactive Oxygen Species

**Figure 7:**
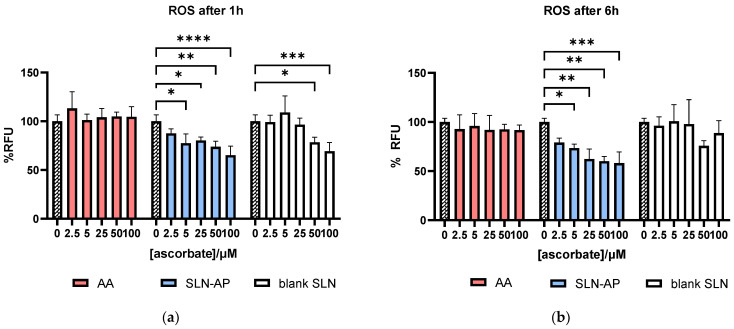
In vitro ROS analysis in cell line U2OS. Cells were treated with AA in free form, SLN-AP and blank SLN for 1 h (**a**) and 6 h (**b**). Fluorescence was measured at 490 nm/570 nm. All treatments were normalized to ascorbate composition. Data are expressed as percent of negative control, mean ± sd, *n* = 3. Two-way ANOVA was performed with Dunnet’s correction. Statistical significance: * *p* ≤ 0.05, ** *p* ≤ 0.01, *** *p* ≤ 0.001, **** *p* ≤ 0.0001.

Analysis of reactive oxygen species has been performed on U2OS cells following 1 h and 6 h of incubation with SLN-AP and blank SLN. Fluorescence is proportional to the amount of ROS detected after the treatment. As visible from Figure 7, AA in free form did not affect ROS both 1 h and 6 h after the treatment. On the other hand, one hour after the treatment, both SLN-AP and blank SLN lower the basal amount of ROS in the U2OS cells, with SLN-AP having significant antioxidative effect when applied in concentrations of 5–100 µM (normalized to ascorbate composition). Six hours after the treatment, SLN-AP still show the antioxidative effect, while blank SLN did not significantly affect the amount of ROS in the cells.

## 4. Discussion

It is widely known that AA and DHA are readily applicable sources of ascorbate because they are soluble in water as well as in the cell culture medium. However, since they are sensitive to light, oxygen and interaction with metal ions, they are easily degraded [1,9]. To overcome this problem, we have decided to use AP as the ascorbate source. As a lipophilic compound, AP should be able to pass the cell membrane [2]. It can be dissolved in water at a very low concentration (179.48 nM at 25 °C), but is readily soluble in higher concentrations in ethanol and some other organic compounds [13]. However, when dissolving AP in ethanol in cell culture medium, AP interacts with various salts and precipitates. To overcome these obstacles, we incorporated AP into the solid lipid nanoparticles.

We prepared two formulations of SLNs, with the only difference being the wt/vol % of Pluronic F-68. We found that this did not have much effect on the zeta potential, as both formulations of the blank SLN had a positive zeta potential (due to the co-surfactant DDAB) and SLN-AP had almost neutral or negative zeta potential when prepared with 3% and 10% Pluronic F-68, respectively. The measured almost neutral and slightly negative zeta potential is likely due to the orientation of the hydrophilic ascorbate on the surface of SLN-AP, which was previously shown by Jukanti et al. for AP liposomes [11]. Formulations prepared with 10% Pluronic F-68 have larger nanoparticles and lower PDI, suggesting it is beneficial for the synthesis of stable formulations. TEM images are consistent with DLS data, however, rod-like shapes are visible in SLN-AP formulation prepared in 3% Pluronic F-68. This is not surprising, as lipids in lipid-based formulations can aggregate into many structures due to varying temperatures, water concentrations, or the presence of different components [14]. For example, Yakimova et al. [15] showed that solid lipid nanoparticles (SLNs) loaded with luminescent markers such as monosubstituted pillar [5] arenes with terminal OH group produced SLNs with elongated shape, i.e., spindle- and rod-shaped SLNs. In our case, incorporation of AP into the formulation may had lead to slight changes in the morphology of SLNs, as shown in Figure 4a.

To evaluate the cellular uptake of the synthesized nanoparticles, we used coumarin-6, a hydrophobic fluorophore commonly used for visualization of drug delivery systems, and incorporated it into the same SLN formulation [16,17]. Zhou et al. [9] and Shi et al. [18] showed the uptake of nanoparticles with coumarin-6 within 4 h; we followed the gradual uptake of SLN-coumarin-6 during 48 h. The fluorescence observed upon visualization of cells incubated with SLN (Figure 5) is partly due to the SLN-coumarin-6 taken up by the cells and partly due to the coumarin-6 released. The gradual increase in fluorescence confirmed the successful uptake of SLN as well as the probable release of the compound contained in the formulation.

When we examined the cytotoxicity of the SLN-AP, we found that U2OS cells are more sensitive to the treatment then HEK 293 cells. Our results show that SLN-AP prepared in 3% and 10% Pluronic F-68 are cytotoxic in much lower concentrations than AA and DHA in the free form. However, overall cytotoxicity is slightly higher for SLNs prepared using 3% Pluronic F-68 (Figure 6) with U2OS cells being more sensitive to the treatment then HEK 293 cells. It is proven that ascorbate can have a selective cytotoxic effect on tumor cells [19,20,21]. Few mechanisms are suggested to explain how can ascorbate have such a selective cytotoxic effect on tumor cells when applied in free form. Ascorbate has a prooxidative effect when interacting with metal ions in cell culture media because of the H_2_O_2_ generation [7,22], it downregulates the transcription factors important for adjusting to hypoxic conditions [23], inhibits GAPDH by generating ROS in *KRAS* and *BRAF* mutant colorectal cancer cells [20] and induces caspase-independent cell death pathway in breast cancer cells [21]. Ascorbate was also investigated when applied in small concentrations together with various chemotherapeutics and was shown to enhance their cytotoxic effect [7]. Several clinical trials are exploring administration of ascorbate in combination chemotherapy [22]. Regarding delivery systems, ascorbate derivative AP is usually explored in combination chemotherapy, where it acts both as a carrier and as a therapeutic—either as an antioxidant [11] or as an enhancer of chemotherapeutic effect [9,10]. Sawant et al. have synthesized AP loaded micelles in order to investigate the effect of AP itself and found that AP loaded micelles have cytotoxic effect due to ROS generation, mostly in the µM range of applied AP [24].

Analysis of reactive oxygen species was performed using the fluorescent probe DCHF-DA, which is de-esterified upon oxidation in the cell [25]. While AA in free form does not affect the level of ROS, SLN-AP significantly decreases them (Figure 7) both 1 h and 6 h after the treatment. Since SLN have antioxidative properties, we conclude that cytotoxicity of SLN is not due to ROS generation. While some studies have reported prooxidant effects of ascorbyl palmitate nanoparticles [18,26], our results show that this formulation can be applied at µM concentrations in which it has no cytotoxic effect and exhibits antioxidant properties both 1 h and 6 h after application. In this formulation, the antioxidant effect of low dose of ascorbate is achieved through a delivery system that should be able to overcome physiological barriers in the organism [27].

## 5. Conclusions

In conclusion, ascorbyl palmitate is a stable source of ascorbate. To ensure successful delivery of AP to the cells, we loaded AP into solid lipid nanoparticles (SLN-AP) using the hot homogenization method with 3% and 10% polymer Pluronic F-68 and their characterization confirmed similar properties. The cytotoxicity assay performed on normal and tumor cell lines showed the appropriate concentration range for the use of both formulations, which depends on whether we want to study antioxidant properties or selective antitumor properties of the SLN-AP. However, both formulations need to be optimized to reduce their intrinsic cytotoxicity.

## Figures and Tables

**Table 1:** Characterization of blank solid lipid nanoparticles (blank SLN) and ascorbyl palmitate-loaded solid lipid nanoparticles (SLN-AP). Before measurement, samples were diluted 10× in ultra-pure water. Average size, polydispersity and zeta potential were measured on Zetasizer, Malvern Panalytical. Data are expressed as mean ± (*n* ≥ 3).

	3% Pluronic F-68		10% Pluronic F-68	
Measurement	Blank SLN	SLN-AP	Blank SLN	SLN-AP
Diameter/nm	18.2 ± 3.21 125 ± 4.52	337 ± 8.52	445 ± 14.3	414 ± 32.1
PDI	0.291 ± 0.0224	0.137 ± 0.0336	0.182 ± 0.0219	0.198 ± 0.0451
zeta potential/mV	+38.1 ± 3.36	+1.45 ± 0.337	+21.5 ± 0.308	−4.18 ± 0.251
